# Archaeal Community Changes Associated with Cultivation of Amazon Forest Soil with Oil Palm

**DOI:** 10.1155/2016/3762159

**Published:** 2016-02-24

**Authors:** Daiva Domenech Tupinambá, Maurício Egídio Cantão, Ohana Yonara Assis Costa, Jessica Carvalho Bergmann, Ricardo Henrique Kruger, Cynthia Maria Kyaw, Cristine Chaves Barreto, Betania Ferraz Quirino

**Affiliations:** ^1^Genomic Sciences and Biotechnology Program, Universidade Católica de Brasília, 70790-160 Brasília, DF, Brazil; ^2^Embrapa Swine and Poultry Research Center, Embrapa, 89700-991 Concórdia, SC, Brazil; ^3^Department of Cellular Biology, Universidade de Brasília, 70910-900 Brasília, DF, Brazil; ^4^Embrapa Agroenergy, 70770-901 Brasília, DF, Brazil

## Abstract

This study compared soil archaeal communities of the Amazon forest with that of an adjacent area under oil palm cultivation by 16S ribosomal RNA gene pyrosequencing. Species richness and diversity were greater in native forest soil than in the oil palm-cultivated area, and 130 OTUs (13.7%) were shared between these areas. Among the classified sequences, Thaumarchaeota were predominant in the native forest, whereas Euryarchaeota were predominant in the oil palm-cultivated area. Archaeal species diversity was 1.7 times higher in the native forest soil, according to the Simpson diversity index, and the Chao1 index showed that richness was five times higher in the native forest soil. A phylogenetic tree of* unclassified Thaumarchaeota* sequences showed that most of the OTUs belong to Miscellaneous Crenarchaeotic Group. Several archaeal genera involved in nutrient cycling (e.g., methanogens and ammonia oxidizers) were identified in both areas, but significant differences were found in the relative abundances of* Candidatus* Nitrososphaera and* unclassified Soil Crenarchaeotic Group* (prevalent in the native forest) and* Candidatus* Nitrosotalea and* unclassified Terrestrial Group* (prevalent in the oil palm-cultivated area). More studies are needed to culture some of these Archaea in the laboratory so that their metabolism and physiology can be studied.

## 1. Introduction

The Amazon forest area represents 50% of the world's remaining rainforests [1]. This biome spreads across Brazil, Bolivia, Peru, Ecuador, Colombia, Venezuela, Republic of Guyana, and French Guyana. The Amazon is the largest Brazilian biome and occupies an area of 4,196,943 km^2^, corresponding to 67% of the Brazilian territory [2]. The Amazon forest provides important ecosystem services such as hydrological cycles and carbon sequestration and storage. More importantly, it hosts over 20% of all plant and animal species in the world [3], indicating its high species diversity.

Amazon's biodiversity encompasses not only macroflora and macrofauna but also its microorganisms, which are often neglected. Mineral materials and organic compounds present in soil create distinct microhabitats populated by different microbial communities. Microorganisms are crucial to the balance of ecosystems, with soil microbial communities playing important roles in soil fertility, plant health, and essential biogeochemical processes such as nitrification, ammonia oxidation, and methanogenesis [4–7].

As of 2010, oil palm was cultivated on 112,500 hectares of land in Brazil [8], primarily in the Amazon region. Oil palm (*Elaeis guineensis* Jacq.) is a highly productive perennial crop, yielding 2,000–8,000 kg oil/ha [8]. The oil, which is extracted from the fruit, has diverse applications in the food and cosmetic industries and can also be used for biodiesel production [9].

Although the Amazon is one of the most species-rich biomes on Earth, little is known about its archaeal diversity. To date only one published microbial ecology study has focused on Amazon soil archaeal diversity using 16S rRNA gene sequencing [10], and only one soil type (i.e., Amazonian dark earth, also called Terra Preta) was studied.

The archaeal taxonomy is a matter of constant change since its proposal, in 1977. Initially two phyla were recognized: Crenarchaeota and Euryarchaeota, but in the subsequent years, many new phyla were proposed. One example is the Thaumarchaeota phylum [11], composed predominantly of mesophilic members; it encompasses the ammonia-oxidizing archaea. The phylum Korarchaeota was proposed in 1996, after the identification of DNA sequences from the Obsidian Pool, in Yellowstone National Park [12], composed of one candidate thermophilic species, whose genome was completely sequenced [13]. Recent works have described putative new phyla, such as Nanoarchaeota, Aigarchaeota, Aenigmarchaeota, Parvarchaeota, and Lokiarchaeota, but these phyla are not widely accepted yet, due to the low number of specimens or DNA sequences available. In addition, further analyses have positioned sequences belonging to these phyla in already described phyla, such as Euryarchaeota or Thaumarchaeota [14–17]. There are some archaeal groups, such as MCG (Miscellaneous Crenarchaeotic Group), which are poorly characterized in terms of phylogenetic affiliation; recent data revealed that this group is probably more closely related to the phylum Thaumarchaeota than to the Crenarchaeota.

Mesophilic archaea seem to play important roles in the cycling of important nutrients such as nitrogen and carbon. The importance of ammonia-oxidizing archaea (AOAs) has been well documented in different ecosystems, such as soils, marine, and freshwater environments, where they sometimes can be found in higher abundance than the ammonia-oxidizing bacteria (AOBs) [18–20]. On the other hand, their real role in nitrification is not yet well understood due to the scarce number of cultured AOAs and the few physiological studies available for this group. The methanogens are among the nonextremophilic archaea, which are widely distributed in anaerobic environments, such as flooded soils, or marine soils and vents. Methanogens are also found in the gut of termites, in rumen of cows, or even in the mouth and intestine of humans. These organisms play important roles in the carbon cycle, transforming small compounds such as acetate and propionate into methane, and removing the hydrogen, which is potentially hazardous to some bacterial cells. On the other hand, methane production is one of the major gases involved in the planet's greenhouse effect (reviewed by [21]).

There are several studies associating land use with changes in the structure and abundance of soil microbial communities, such as the influence of the land use over the diversity of AOA and AOB in grassland soils [22] and the impacts of edaphic factors on those archaea in tropical soils [23]. Therefore, the conversion of native forest into palm tree culture can be another example of a potential impact of the oil palm cultivation in the archaeal communities of Amazonian soils.

This work aimed to improve our understanding of how the soil archaeal community is impacted by oil palm cultivation. To this end, microbial DNA was extracted from soil samples from native forest and an adjacent area under oil palm cultivation. The archaeal 16S ribosomal RNA (rRNA) gene was amplified and sequenced using high-throughput methods for comparative analysis. Here we show for the first time that soil archaeal diversity is reduced in soil under oil palm cultivation compared to native forest soil.

## 2. Materials and Methods

### 2.1. Site Description, Sampling, and Processing

**Figure 1:**
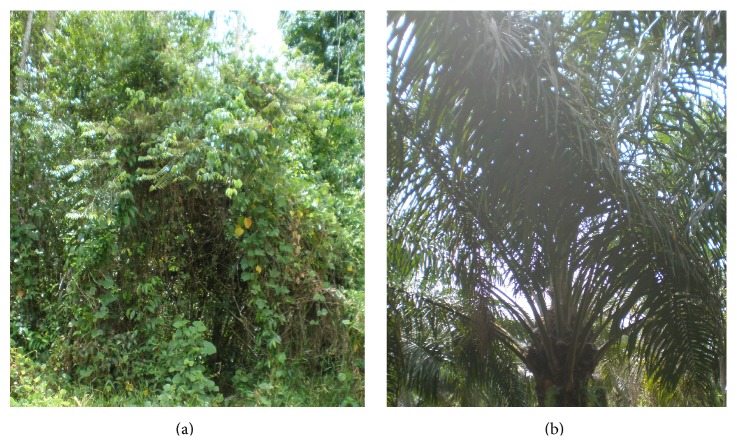
(a) Native forest and (b) oil palm-cultivated sites.

Soil samples were collected in the State of Pará, Brazil, in an oil palm-cultivated area and an adjacent area of Amazon native forest near the city of Moju (Figure 1). The tropical forest in this region is dense, with trees that are 25–35 m tall [24]. The climate is equatorial, hot, and humid (Ami type according to the Köppen climate classification). Annual temperatures range from 25°C to 27°C, and rainfall is 2,000–3,000 mm per year being irregularly distributed [25]. The soil is predominantly “Latossolo Amarelo” (a type of Oxisol) [26].

The oil palm cultivation in the sampled farm is not as controlled as other crops (Figure 1(b)). There is no irrigation regime; natural precipitation of the rainforest is the only way these plants are irrigated. In the Amazon, the soil is very moist, due to the high precipitation levels during the year. In the studied area, the annual period of flooding is from February to April. Furthermore, the soil around the palm trees is not fertilized in a homogeneous fashion, since only one side of the plants is directly fertilized.

In October 2010, after plant litter was removed, a soil borer was used to obtain four 10 cm deep soil samples from three points in the oil palm-cultivated area (S02°00′28.9′′/W048°37′57.4′′, S02°00′29.2′′/W048°37′56.6′′, and S02°00′31.3′′/W048°37′54.3′′) (Figure 1(b)) and four samples from the native Amazon forest area (S02°00′27.2′′/W048°35′53.0′′) (Figure 1(a)). The samples collected in each area were mixed, ground, and sieved to remove larger particles, yielding one composite sample for each area, with approximately 1 kg each. The samples were stored in plastic bags on dry ice during transportation. A subsample was sent to physicochemical analysis at SoloQuímica Análises de Solo Ltda. (Brasília, DF, Brazil). The rest of the samples were then stored at −80°C until DNA extraction. Initially, the physicochemical characteristics of the soils samples were evaluated individually and a high variation among replicas was observed. This result was due to the heterogeneous fertilization of the palm trees in the cultivation fields in Amazon; therefore, composite samples were necessary to describe the microbiota in oil palm soil.

### 2.2. DNA Extraction, PCR, and Pyrosequencing Analysis

Total DNA was extracted according to the protocol of Smalla et al. [27], using 2 g soil per sample. To minimize DNA extraction bias, this procedure was performed in quadruplicate. PCR reactions were performed using the following primers specific for Archaea: 340F (5′-CCC TAY GGG GYG CAS CAG-3′) and 1000R (5′-GGC CAT GCA CYW CYT CTC-3′) [28]. Archaea 16S rRNA genes were amplified, yielding 660 bp amplicons. Adapters used as priming sites for both amplification and sequencing (454 Life Sciences, Branford, CT, USA) were ligated to the 5′ end of the primer sequences. Each 20 *μ*L PCR reaction contained 10–30 ng total DNA, 1x reaction buffer, 4 *μ*M dNTP, 10 *μ*M of each primer, 200 *μ*g/mL bovine serum albumin, 0.5 U KAPA2G Robust HotStart polymerase, and Milli-Q water. Amplification was performed in an Applied Biosystems GeneAmp® PCR System 9700 Thermal Cycler (Applied Biosystems, Foster City, CA, USA) using the following program: 2 min at 98°C; followed by 30 cycles of 30 seconds at 95°C, 30 seconds at 57°C, and 1 minute and 30 seconds at 72°C; and a final step consisting of 7 minutes at 72°C. To minimize PCR bias, at least four PCR amplification reactions per sample were pooled before purification using the GeneJET PCR Purification Kit (Thermo Fisher Scientific, Waltham, MA, USA). After quantification using a Qubit® fluorometer (Invitrogen, Carlsbad, CA, USA), NanoDrop*™* 1000 Spectrophotometer (Thermo Fisher Scientific), and 2% agarose gel with ethidium bromide, DNA samples were sent for pyrosequencing (GS FLX Titanium Platform at Macrogen, South Korea). A total of 592,145 reads greater than 500 bp were obtained in three-quarters of a plate: 49,314 reads from the oil palm-cultivated soil and 542,831 reads from the native forest soil.

### 2.3. Data Analysis

All 16S rRNA gene pyrosequencing reads were analyzed using the original standard flowgram format (SFF) output file from the sequencer in MOTHUR, version 1.33.3 [29], for the removal of short sequences, sequences with errors, low-quality sequences, chimeras, and possible contaminants. After this removal, a total of 39,111 sequences for oil palm-cultivated area and 436,532 sequences for native forest were obtained. Further analysis was performed after normalizing the number of sequences per sample to the lowest number of high-quality sequences (i.e., 39,111 sequences in the oil palm-cultivated area sample).

All 16S rRNA gene reads were analyzed using MOTHUR for taxonomic classification following the 454 standard operating procedure available online (http://www.mothur.org/wiki/454_SOP) [30]. The PyroNoise algorithm [31] was used to denoise the flowgram file (removal of barcodes and quality filtering). The SILVA database (release 119) was used for sequence alignment and clustering based on 97% sequence similarity (0.03 genetic distance), using the nearest neighbor method [32]. The UCHIME algorithm [33] was used for detection and removal of chimeric reads; this function was implemented in MOTHUR and performed without a reference database. The one-gap method in MOTHUR was used to calculate the pairwise distance matrix. Archaeal taxonomic classifications of each representative OTU were performed in MOTHUR, using the SILVA database (release 119) clustered at 97% sequence similarity [32]. This classification was used to estimate the relative abundance of reads per genus. Samples were randomly chosen for subsampling and normalized to the lowest number of sequence reads obtained. Chao1 [34], abundance-based coverage estimator (ACE) [35], Shannon diversity index [36], and Good's coverage [37] were then used to estimate alpha diversity. MOTHUR was also used to perform all statistical analyses. A phylogenetic tree was created using all Thaumarchaeota OTUs from each sample that were unclassified at the class level. A new tree was created with sequences randomly chosen by MOTHUR that were deemed representative of the entire set. Data were analyzed using the neighbor-joining method with the Jukes-Cantor correction and 1,000 bootstrap replicates in MEGA6 [38]. In addition, OTUs with 3% dissimilarity were selected by MOTHUR to avoid redundancies in the phylogenetic tree. The chosen sequences were aligned with 16S rRNA gene sequences from GenBank [39] representing the main Archaea groups:* Methanosarcina baltica* (AB973356),* Methanoregula formicica* (NR112877),* Methanocella paludicola* (NR074192),* Halococcus hamelinensis* (LN651155), and* Pyrococcus furiosus* (U20163) from the phylum Euryarchaeota;* Pyrobaculum aerophilum* (NR102764) from the phylum Crenarchaeota;* Candidatus* Nitrososphaera sp. (FR773157) and* Candidatus* Nitrososphaera gargensis (GU797786) from Thaumarchaeota Group I.1b;* Nitrosopumilus maritimus* (JQ346765) and* Cenarchaeum symbiosum* (U51469) from Thaumarchaeota Group I.1a;* uncultured acidic red soil AOA *(FJ174727),* uncultured trembling aspen archaeon* (EF021427), and* boreal forest archaeon* (X96688) from Thaumarchaeota Group I.1c; and several uncultured organisms classified according to the SILVA database as Thaumarchaeota Miscellaneous Crenarchaeotic Group (FR745121, AM910782, JX984848, KC510333, FJ485299, KC831395, FJ920714, FJ485307, HM051127, HM051130, and HM051125). A 16S rRNA gene sequence from Acidobacteria KBS 96 (FJ870384) was used as an outgroup. A second phylogenetic tree was created using only Euryarchaeota OTUs from each sample that were unclassified at the class level, with the same parameters and reference sequences.

A Venn diagram was generated by grouping OTUs clustered at 97% similarity to show the number of OTUs unique to each area and those shared between samples.

Differences in taxonomic distribution of archaea between native forest and the oil palm-cultivated area were evaluated by Fisher's exact test (*q* ≤ 0.05) using Statistical Analysis of Metagenomic Profiles (STAMP) software [40]. The confidence interval was estimated using the asymptotic method [41], and correction of the *q* value was calculated using Storey's false discovery rate (FDR) approach [42]. A filter was applied to show only the differences between proportions above 0.5%.

## 3. Results

Good's coverage estimate of the completeness of sampling (Table 1) was higher for the oil palm-cultivated area than for the native forest.

**Figure 2:**
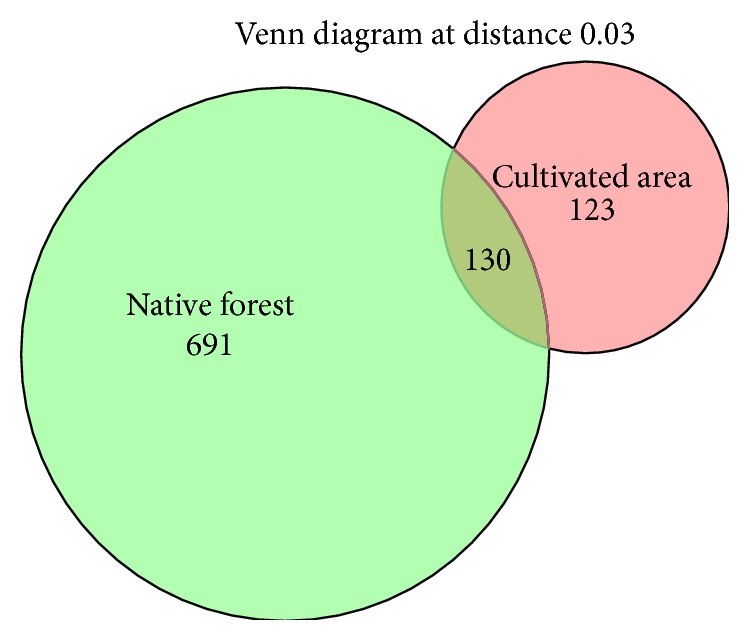
Venn diagram depicting the number of shared and unique operational taxonomic units (OTUs) in native forest and oil palm-cultivated area.

The number of archaeal OTUs was higher in the native forest soil (821 sequences) than in soil under oil palm cultivation (253 sequences) (Figure 2), with 130 OTUs (13.8%) shared between the two areas. Archaeal species richness, as assessed by abundance-based estimators, Chao1 and ACE, was higher in the native forest than in the oil palm-cultivated area. Similarly, the Inverse Simpson and Shannon indices indicated that archaeal species diversity was higher in native forest soil compared to soil under oil palm cultivation (Table 1).

Soil physicochemical parameters differed between the areas (Table 2), with higher levels of phosphorous, potassium, and total carbon in the oil palm-cultivated soil and higher levels of calcium and organic matter in the native forest soil.

**Figure 3:**
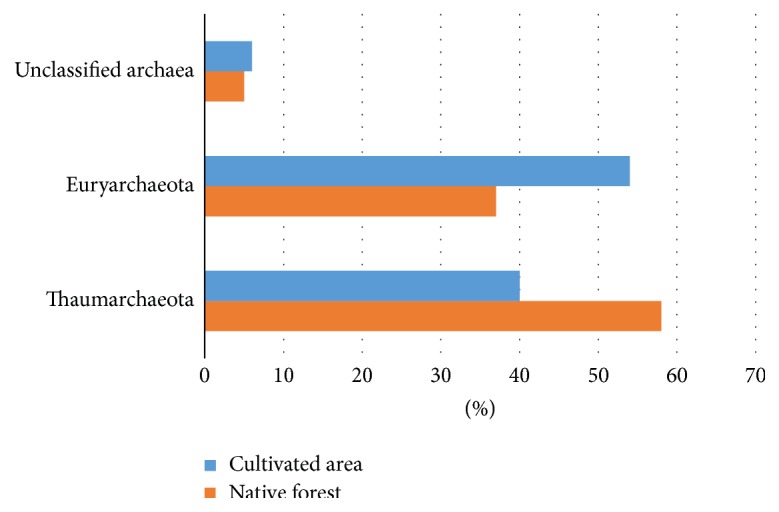
Comparison of archaeal phyla present in soil samples from native Amazon forest (orange) and an area under oil palm cultivation (blue). The percentages of unclassified sequences are shown.

In both areas, only two archaeal phyla (Thaumarchaeota and Euryarchaeota) were detected (Figure 3). Thaumarchaeota were predominant in the native forest soil (58%), and Euryarchaeota were predominant in the oil palm-cultivated soil (54%). The percentage of unclassified archaea was similar in both soils (native forest, 5%; oil palm-cultivated area, 6%).

**Figure 4:**
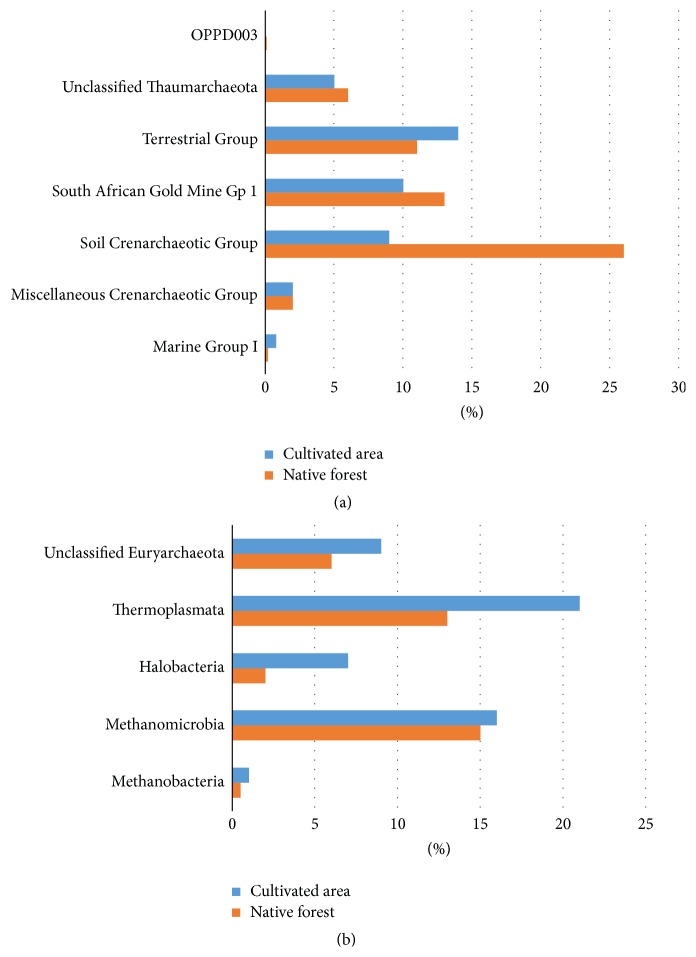
Comparison of archaeal classes present in soil samples from native Amazon forest (orange) and an area under oil palm cultivation (blue). Classes within the phyla Thaumarchaeota (a) and Euryarchaeota (b) are shown, as well as the percentage of unclassified sequences.

At the class level, a total of 10 groups were detected in both samples: Halobacteria, Methanobacteria, Methanomicrobia, and Thermoplasmata in the phylum Euryarchaeota; and Marine Group I, OPPD003, Soil Crenarchaeotic Group, South African Gold Mine Gp 1, Terrestrial Group, and Miscellaneous Crenarchaeotic Group in the phylum Thaumarchaeota (Figures 4(a) and 4(b)). The most abundant class present in native forest soil was the Soil Crenarchaeotic Group, representing 26% of all native forest OTUs (Figure 4(a)) and 45% of the Thaumarchaeota sequences (not shown), followed by Methanomicrobia, representing 15% of all native forest OTUs (Figure 4(b)) and 42% of the Euryarchaeota sequences (not shown). The most abundant class present in the oil palm-cultivated area was Thermoplasmata, representing 21% of all cultivated area OTUs (Figure 4(b)) and 39% of the Euryarchaeota sequences (not shown), followed by Methanomicrobia, representing 16% of all cultivated area OTUs (Figure 4(b)) and 30% of all Euryarchaeota sequences (not shown). The predominant class from the phylum Thaumarchaeota in the oil palm-cultivated area was Terrestrial Group, representing 14% of the total OTUs (Figure 4(a)) and 35% of all Thaumarchaeota sequences (not shown).

**Figure 5:**
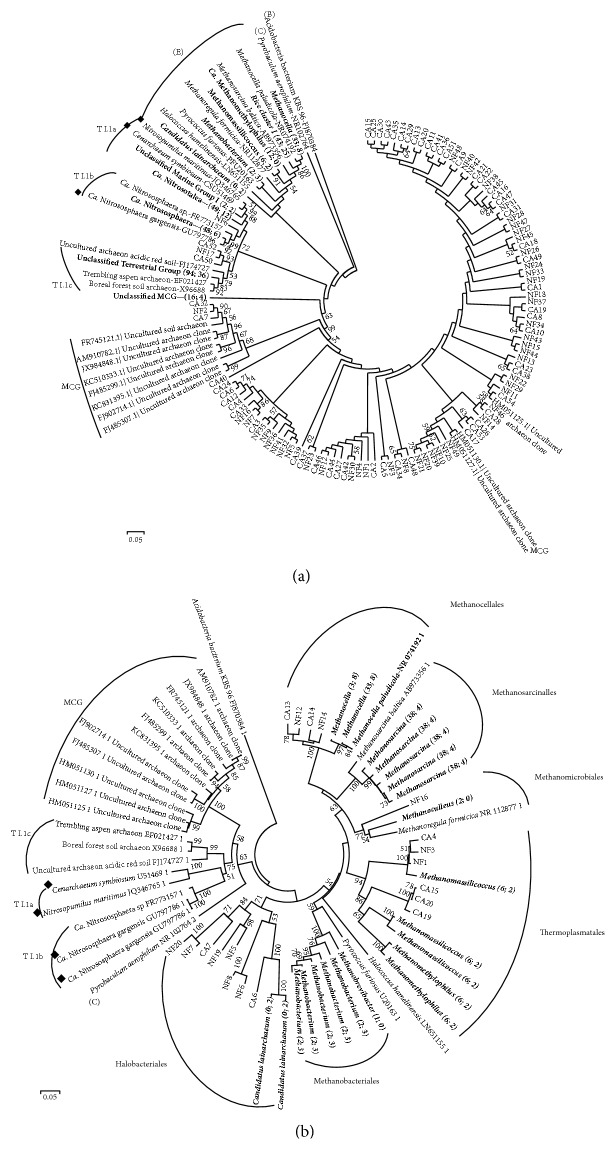
Phylogenetic trees of (a) unclassified Thaumarchaeota 16S rRNA gene sequences from native forest soil and soil under oil palm cultivation and (b) 16S rRNA gene sequences classified as Euryarchaeota from native forest soil and soil under oil palm cultivation. The neighbor-joining method and Jukes-Cantor corrections were used with 1,000 bootstrap replicates. Bootstrap values above 50 are shown in the trees. A representative number of operational taxonomic units (OTUs) were selected at 3% dissimilarity by MOTHUR to avoid redundancies in the phylogenetic trees. Randomly chosen OTUs from native forest soil and from soil under oil palm cultivation were used in the construction of the trees, along with archaeal reference sequences (from GenBank) from the following groups: Acidobacteria (B), Crenarchaeota (C), Euryarchaeota (E), Miscellaneous Crenarchaeotic Group (MCG), Thaumarchaeota Group I.1a (T I.1a), Thaumarchaeota Group I.1b (T I.1b), and Thaumarchaeota Group I.1c (T I.1c). The AOAs are highlighted with the symbol *◆*. Sampling site is indicated by “NF” (native forest) or “CA” (oil palm-cultivated area), followed by the number of the OTUs (NAxx or CAxx). OTU sequences classified to at least the class level are shown in bold, followed by two numbers in parentheses indicating the number of sequences of that OTU detected in native forest soil and the number detected in the oil palm-cultivated area. The* scale bar* represents the 5% estimated sequence divergence.

Because it is a newer phylum, Thaumarchaeota have few cultivated representatives compared with Euryarchaeota and fewer sequences in databases such as SILVA. To determine how the Thaumarchaeota sequences that could not be classified at the genus level were related to each other, a phylogenetic tree was constructed with representative sequences (Figure 5(a)). One native forest OTU clustered with Group I.1b (NF6), and one native forest OTU (NF17) and two cultivated area OTUs (CA50, CA52) clustered with Group I.1c. The remaining OTUs formed different clusters, all associated with the Miscellaneous Crenarchaeotic Group. Most unclassified Thaumarchaeota sequences from the native forest and oil palm-cultivated area grouped together, although a few unclassified OTUs from the cultivated area (e.g., CA51 to CA15 and CA24 to CA33) and native forest (e.g., NF21 to NF49 and NF5 to NF35) formed separate clusters.

The unclassified sequences at genus level in the Euryarchaeota phylum were all related to the orders Methanocellales, Methanosarcinales, Methanomicrobiales, Thermoplasmatales, Methanobacteriales, and Halobacteriales (Figure 5(b)). The classified sequences at genus level were* Methanocella, Methanosarcina, Methanoculleus, Methanomassiliicoccus, Methanomethylophilus, Methanobrevibacter, Methanobacterium,* and* Candidatus* lainarchaeum (Figure 5(b)).

**Figure 6:**
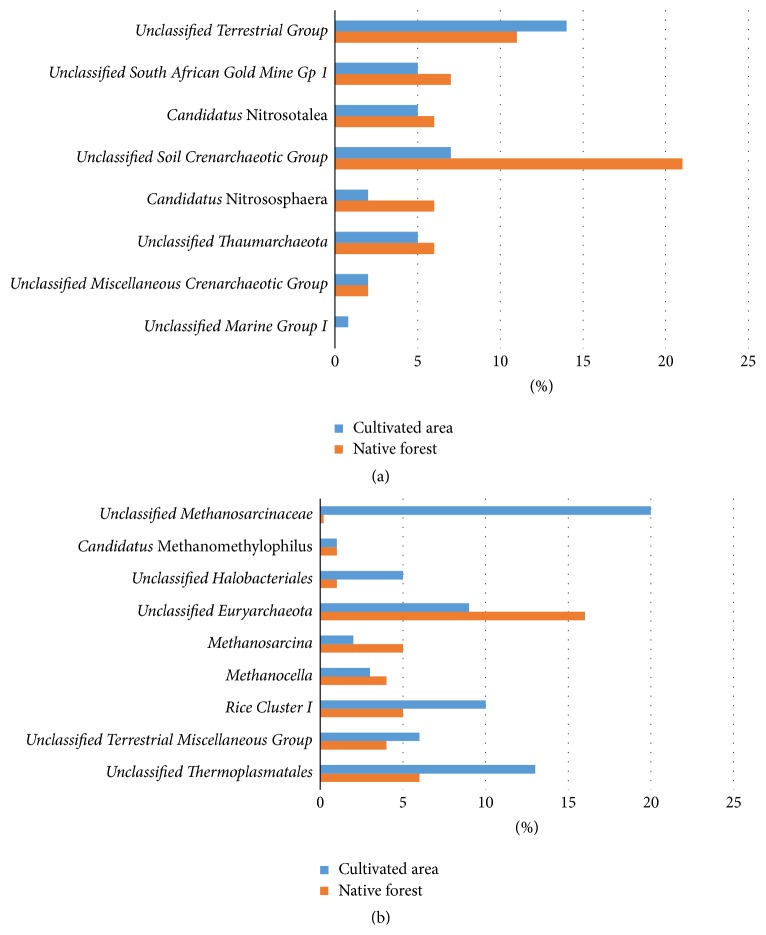
Percentage of archaeal operational taxonomic units within the Thaumarchaeota (a) and Euryarchaeota (b) phyla classified at the genus level for native forest soil (orange) and soil under oil palm cultivation (blue). The percentage of unclassified sequences at genus level is shown.

The genus-level analysis showed that* unclassified Soil Crenarchaeotic Group* was predominant in the native forest, accounting for 21% of all native forest OTUs (Figure 6(a)). In the oil palm-cultivated area the predominant genus was* Unclassified Terrestrial Group*, accounting for 14% of all cultivated area OTUs (Figure 6(b)).

A total of 31 groups of OTUs were identified (only major groups are shown), most of which are unclassified. The classified groups in the phylum Euryarchaeota are* Candidatus* lainarchaeum,* Methanobacterium*,* Methanobrevibacter*,* Methanocella*,* Rice Cluster I*,* Methanoculleus*,* Methanoregula*,* Methanimicrococcus*,* Methanosarcina*,* Candidatus* Methanomethylophilus, and* Methanomassiliicoccus* (Figure 6(b)). The classified groups in the phylum Thaumarchaeota are* Candidatus* Nitrososphaera and* Candidatus* Nitrosotalea (Figure 6(a)).

**Figure 7:**
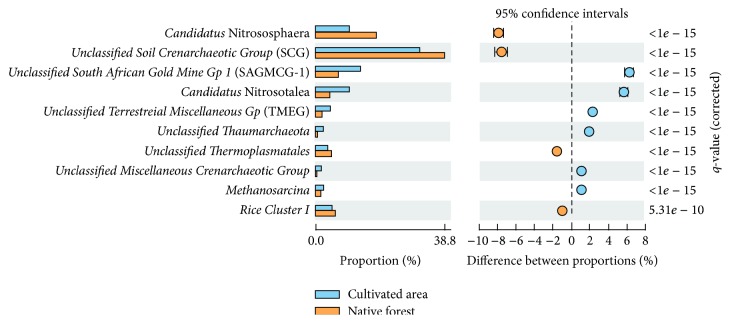
Archaeal genera that differ significantly (*q* < 0.05) in relative abundance between native forest soil (orange) and soil under oil palm cultivation (blue). For the purpose of statistical analysis performed using STAMP software, the sum of the percentages of archaea classified at the genus level in both soils is 100%. Differences between proportions of each genus are shown with 95% confidence intervals. Unclassified sequences were used to calculate frequency profiles but are not displayed.

The analysis of the genera that differ significantly in relative abundance between native forest soil and the oil palm-cultivated area revealed that the greatest difference was seen for* Candidatus* Nitrososphaera (Figure 7). This genus was predominant in the native forest soil, accounting for 18.2% of the sequences, but represented only 10.3% of the sequences from the oil palm-cultivated area.* Unclassified Soil Crenarchaeotic Group* was also predominant in the native forest (i.e., 38.8% of sequences in the native forest and 31.2% of sequences in the oil palm-cultivated area), whereas* unclassified South African Gold Mine Gp 1* was predominant in the oil palm-cultivated area (i.e., 7.3% of sequences in the native forest and 13.5% of sequences in the oil palm-cultivated area).* Candidatus* Nitrosotalea represented 4.4% of the native forest sequences and 10.0% of the oil palm-cultivated area sequences. The relative abundance of* unclassified Terrestrial Miscellaneous Group*,* unclassified Thaumarchaeota*,* unclassified Thermoplasmatales*,* unclassified Miscellaneous Crenarchaeotic Group*,* Methanosarcina*, and* Rice Cluster I* also differed significantly between native forest soil and soils under oil palm cultivation, but these differences were smaller.

## 4. Discussion

The Amazon forest is known for its macrospecies diversity; however, few studies have addressed its microbial diversity. Among the domains of life, Archaea are undoubtedly the less well known.

In this work almost 600,000 archaeal 16S rRNA gene sequences were obtained from native forest soil and an adjacent area under oil palm cultivation. After processing (i.e., removal of short sequences, sequences with errors, low-quality sequences, chimeras, and possible contaminants) and normalization, 39,111 sequences per area remained for analysis. This number of sequences provided sufficient coverage of archaeal diversity in both areas (Table 1). The primers used were based on a large number of 16S rRNA sequences from the SILVA database [28]; however, given that little is known about this domain, it is unlikely that the archaeal diversity was fully covered [43].

The main physicochemical differences between soils of the native forest and the oil palm-cultivated area were the levels of phosphorous, potassium, calcium, organic matter, and total carbon (Table 2). Correlations between phylogeny and physicochemical parameters were evaluated using PCA and NMDS; however no clear clusters or correlations were observed (results not shown).

In a study of the long-term effects of inorganic fertilizers on microbial communities, Zhong and Cai [44] reported that phosphorus application indirectly altered microbial parameters in soils and increased crop yields by elevating the levels of organic matter. Wessen and collaborators used real-time PCR quantification to show the impacts of fertilizer use on several bacterial phyla and the archaeal phylum Crenarchaeota (Thaumarchaeota Group I.1c, after reanalysis using the SILVA database 119) [45]. They reported that bacterial and archaeal classes respond differently to fertilization, primarily because of changes in certain soil parameters such as pH, total nitrogen, and the carbon: nitrogen ratio. In our study levels of total carbon and organic matter were higher in the native forest than in the oil palm-cultivated area, which may have contributed to higher archaeal diversity. Although there are no studies on the effect of total soil carbon on archaeal communities, there are studies on the bacterial communities in Amazonian soils. The work of Edgar et al. [33] described distinct soil bacterial communities under different vegetation types. The authors found that bacterial diversity correlated with total organic carbon and total nitrogen content [46]. Furthermore, in a study of bacterial diversity and microbial biomass in forest, pasture, and fallow soils in the Amazon basin, microbial biomass was highest in pasture soils, which also had 30%–47% higher carbon levels than the other sites over the course of a year [47].

Several studies have also reported alterations in the composition of archaeal [48], bacterial [47], and fungal [49] communities associated with land use changes. A study of archaeal communities in Amazonian anthrosols under various land uses (agriculture, pasture, and secondary forest) and cultivation practices (e.g., eggplant, banana, citrus, and manioc) [10] reported that agriculture negatively affected archaeal community diversity. Results of ribosomal intergenic spacer analysis showed that soil type is also responsible for changes in archaeal communities in soils under different types of land use (native grassland, native forest,* Eucalyptus* and* Acacia* plantations, and soybean and watermelon fields) [50].

The Venn diagram showed that the native forest soil has a greater number of unique sequences (Figure 2). This was not surprising, due to the greater archaeal richness and diversity observed in the native forest soil (Table 2). The number of unique OTUs found in the oil palm-cultivated area is almost the same as that of shared OTUs with the native forest area. Although oil palm cultivation decreased archaeal diversity in the soil, there is still a core group of archaea OTUs shared with the native forest, suggesting that these archaea have certain characteristics that allow them to persist after the change in land use. It cannot be ruled out that these archaea are metabolically inactive. The smaller number of unique sequences in the oil palm-cultivated area may be related to a lower complexity of the vegetation (i.e., oil palm monoculture versus great plant diversity in the forest), since plant diversity could affect these communities [51].

In our study, all OTUs identified belong to the domain Archaea and were classified into two phyla, Euryarchaeota and Thaumarchaeota. It is important to mention that the databases were only recently updated to recognize the phylum Thaumarchaeota; therefore, previous studies on Archaea could not recognize this phylum, as proposed by Brochier-Armanet et al. [11]. The creation of this novel phylum was supported by a study comparing the genomes of two marine ammonia-oxidizing archaea (AOAs) that identified a core set of informational genes specific to this group [52]. Therefore, to allow comparisons with other studies, we reanalyzed their data whenever possible, updating the taxonomic classification to include the phylum Thaumarchaeota.

The work of Chao and Shen [35] focused on the impacts of land use (i.e., agricultural systems of indigenous people and cattle pasture) on archaeal communities by PCR (*amoA* gene) and denaturing gradient gel electrophoresis analysis of different Western Amazon soils. The databases at the time did not consider Thaumarchaeota in their classification; however, our reanalysis of their dataset using an updated database showed that Euryarchaeota and Thaumarchaeota were present in their samples along with Crenarchaeota (Thermoprotei). In addition, changes were observed in the structure of soil archaeal communities when comparing the cultivated areas with forest areas that had been converted to pasture.

In our study, differences in the percentages of Thaumarchaeota and Euryarchaeota found in the soil samples were not striking. Thaumarchaeota were predominant in the native forest (58%), whereas Euryarchaeota were predominant in the oil palm-cultivated area, with 54% (Figure 3). This result is consistent with other comparative studies on archaeal communities in different types of Brazilian soils (i.e., Amazonian anthrosols, Caatinga, and Cerrado) [10, 53, 54] and a study of different tropical forest soil types that reported Thaumarchaeota as the predominant phylum [44].

The phylum Thaumarchaeota comprises all known AOAs and is directly involved in nitrogen metabolism. Ammonia is used as an energy source by Group I.1a, found predominantly in aquatic systems [52], and Group I.1b, found in soils [55]. Thaumarchaeota Group I.1c is primarily found in acidic soils, but the ecological roles of these archaea are still unknown [56, 57].

The literature presents conflicting data about the proportion of AOAs/AOBs soils. In some studies, AOAs are predominant [58], while in others, AOBs are more abundant [59]. However, several studies found a predominance of the archaeal* amoA* gene over the bacterial* amoA* gene in soils [60]. AOAs are frequently detected in soils, their abundance range from 0.5% to 10% of the prokaryotic community, and their abundance is strongly influenced by physicochemical conditions as well as the organic matter content [61, 62]. In acidic soils, AOAs appear to have a more important role in ammonia oxidation than AOBs [57].

The Soil Crenarchaeotic Group, a class within Thaumarchaeota according to the SILVA database classification, was detected in both soil samples, but a higher representation was observed in native forest soil. This class was discovered in boreal forest soil [63].* Candidatus* Nitrososphaera, which belongs to the Soil Crenarchaeotic Group class, is a member of Thaumarchaeota Group I.1b; it is an AOA that uses ammonia or urea as an energy source [64, 65].

On the other hand, members of the phylum Euryarchaeota are directly involved in carbon metabolism, with several classes of methanogens and methane-oxidizing organisms [66–68]. Navarrete et al. [48] studied the impact of converting Amazon native forest to pasture and reported changes not only to the composition and abundance of soil microbial communities but also to a decrease in the diversity of gene function.

Within the phylum Euryarchaeota, Methanomicrobia and Thermoplasmata were the main classes detected in our samples, along with a few representatives of Methanobacteria and Halobacteria. Members of Methanobacteria and Methanomicrobia are methanogens [69]. Methanomicrobia were present in both native forest soil and oil palm-cultivated soil, with larger representation in the oil palm-cultivated area. Within Methanomicrobia, the genera* Rice Cluster I*,* Methanosarcina*, and* Methanocella* were present in both samples (Figure 6(b)). These genera are closely related and found in rice fields that produce methane [70]. The order Methanomicrobiales was detected only in the native forest soil, which can probably be explained by the relatively high levels of carbon and organic matter. This archaeal order is responsible for the final step of methane production in the carbon cycle by anaerobic degradation of organic matter [66, 71]. However, the contribution of the Methanomicrobia to the carbon cycle in the oil palm-cultivated soils could not be assessed since there are no data on the methane production in the studied areas.

Among the Thaumarchaeota sequences used to generate the phylogenetic tree that were unclassified at the genus level, several OTUs formed different clusters associated with the Miscellaneous Crenarchaeotic Group (Figure 5(a)). These sequences were from both soil samples, indicating that the unclassified Thaumarchaeota in the native forest and the oil palm-cultivated area are phylogenetically related.

Most of the sequences remained unclassified at the genus level for both Thaumarchaeota and Euryarchaeota (Figures 6(a) and 6(b)). This result likely reflects the fact that most taxonomic databases do not have many representatives of Archaea, especially belonging to the phylum Thaumarchaeota; thus few classifications of new sequences reach the genus level. Several genera were identified as* Candidatus* according to the International Code of Nomenclature of Bacteria, indicating that characterization of the cultured prokaryote is incomplete [72].

The proportions of only four groups classified at the genus level differed significantly between native forest and oil palm-cultivated soils (*Methanosarcina* and* Rice Cluster I* in the phylum Euryarchaeota and* Candidatus* Nitrososphaera and* Candidatus* Nitrosotalea in the phylum Thaumarchaeota) (Figure 7). With respect to the methanogens, sequences related to* Methanosarcina* were slightly more abundant in soil under oil palm cultivation (Figure 7), whereas sequences belonging to the* Rice Cluster I* were more abundant in native forest soil.

In the Thaumarchaeota phylum,* Candidatus* Nitrososphaera, an AOA member of group I.1b [65], was detected in both samples but had a much higher representation in native forest (native forest, 18.2%; oil palm-cultivated area, 10.3%) (Figure 7). In a comparative study of the archaeal communities in Amazonian dark earth (“Terra Preta”) and adjacent soils,* Candidatus* Nitrososphaera was the main archaeal group found in both soils [10]. In contrast,* Candidatus* Nitrosotalea, another AOA, was more abundant in the oil palm-cultivated area (10.0%) than in native forest soil (4.4%) in our study (Figure 7). Lehtovirta-Morley et al. [73] cultivated the obligate acidophilic ammonia oxidizer* Candidatus* Nitrosotalea devanaterra from a nitrifying acidic soil. Later, the same group characterized two strains of acidophilic AOA belonging to this genus from a Chinese paddy field and a Scottish agricultural soil [74]. In our study the greater representation of* Candidatus* Nitrososphaera and* Candidatus* Nitrosotalea in native forest and oil palm-cultivated soils, respectively, can be explained by the low soil pH (native forest, pH 5.50; oil palm-cultivated area, pH 4.83), since in basic soils the activity and growth of these archaea are diminished by the exponential decrease in nitrite [6, 75]. In addition, soil under oil palm cultivation is fertilized with nitrogen, which may explain the higher level of* Candidatus* Nitrosotalea in this area compared with that of native forest soil. Finally, although the cultivated members of the genus* Ca.* Nitrososphaera grow better on neutral pH, they have been reported to grow on pH values ranging from 6.0 to 8.5. In addition, a recent work published by Wang et al. (2014) [76] described active AOAs from an acidic soil (pH 4.92) which are closely related to Nitrososphaera, known as Nitrososphaera-like organisms by molecular phylogenetic methods. Most of our sequences were classified as Nitrosotalea-like, which are sequences belonging to the group I.1a. This group is typically found in aquatic environments, such as lake sediments, or marine environments. A similar ubiquitous distribution in soils is found for the group I.1b, which are not restricted to a specific soil pH condition.

## 5. Conclusions

Although archaea are known to be ubiquitous, there is still much to be learned about their diversity, biological roles, and metabolic capabilities. This work is the first to compare archaeal communities associated with the Amazon forest soil with those of soil under oil palm cultivation.

Archaeal diversity was found to be lower in oil palm-cultivated soil than in the native forest soil. Only about 30% of the OTUs were found in the oil palm soil, in comparison to the native forest. However, two main phyla were detected in both samples, Thaumarchaeota and Euryarchaeota, with a predominance of Thaumarchaeota in native forest soil. These results support previous studies in other Brazilian biome native soils (e.g., Caatinga, Cerrado) as well as soil under different types of land use (e.g., pasture and agricultural). It is also important to mention that there are also groups of uncertain classification, associated with the Crenarchaeota phylum.

Although knowledge about Archaea has increased dramatically in the last few decades, little is known about the biology of many archaeal groups, and the taxonomy of Archaea is still a work in progress. Thus, it is not surprising that many sequences identified in this work remain unclassified, sometimes even at higher taxonomic levels. It is not clear whether the relatively small overall difference between soil archaeal communities of the Amazon forest and oil palm-cultivated land is a consequence of the current state of archaeal taxonomy or whether it indicates that archaeal diversity is lower than bacterial diversity [77].

It is also important to consider that with land use change, due to agricultural practices, the microstructure of the soil is broken down, which can reduce the natural habitat fragmentation, allowing potential interactions among the resident microbiota that otherwise would not happen [78, 79].

A phylogenetic tree of unclassified Thaumarchaeota 16S rRNA gene sequences was useful in exploring relationships among these sequences. When taxonomic classification of sequences was possible, it revealed the presence of OTUs from genera known to be involved in the metabolism of carbon and nitrogen (methanogens* Methanocella* and* Methanosarcina*; AOA* Candidatus* Nitrososphaera and* Candidatus* Nitrosotalea), hinting at possible roles for these archaeal communities in Amazon soils.

More studies are needed to elucidate these roles. Culturing some of these archaea in the laboratory would allow their metabolism and physiology to be studied in detail, allowing for a better understanding of the roles of Archaea in the biogeochemical cycles.

## Figures and Tables

**Table 1:** Number of operational taxonomic units (OTUs), Good's coverage estimator, OTU richness indices (Chao1, ACE), and Inverse Simpson and Shannon diversity indices for archaeal communities in native forest soil and soil under oil palm cultivation.

Sample	OTUs^*∗*^	Good's coverage (%)	Chao1	ACE	Inverse Simpson	Shannon
Native forest	821	98.71	2,124	4,645	26.45	4.22

Oil palm-cultivated area	253	99.72	424	674	15.47	3.31

^*∗*^Considering 97% sequence similarity in MOTHUR.

**Table 2:** Physicochemical properties of native forest soil and soil under oil palm cultivation.

	Native forest	Oil palm-cultivated area
pH_(in water)_	5.50	4.83
P (mg·dm^−3^)	1.40	70.70
K (mg·dm^−3^)	0.03	0.11
Ca^+2^ (cmol·dm^−3^)	1.40	0.33
Mg^+2^ (cmol·dm^−3^)	0.10	0.17
Al^+3^ (cmol·dm^−3^)	0.40	0.73
H + Al (cmol·dm^−3^)	6.20	7.27
Na^+^ (cmol·dm^−3^)	0.01	0.02
Organic matter (g/kg)	31.00	11.63
Total C (g/kg)	18.00	6.77
Total N (mg/kg)	N/A^#^	1.15
NO_3_^−1^ (mg/kg)	N/A^#^	0.79
NO_2_^−1^ (mg/kg)	N/A^#^	0.14
NH_4_^+^ (mg/kg)	N/A^#^	0.21

^#^Data not available.
